# Molecular cloning and RNA interference-mediated functional characterization of a Halloween gene *spook* in the white-backed planthopper *Sogatella furcifera*

**DOI:** 10.1186/1471-2199-14-19

**Published:** 2013-09-04

**Authors:** Shuang Jia, Pin-Jun Wan, Li-Tao Zhou, Li-Li Mu, Guo-Qing Li

**Affiliations:** 1 Education Ministry Key Laboratory of Integrated Management of Crop Diseases and Pests, College of Plant Protection, Nanjing Agricultural University, Nanjing 210095, China

**Keywords:** *Sogatella furcifera*, Halloween gene, Ecdysteroidogenesis, RNA interference, Lethality, Development

## Abstract

**Background:**

Ecdysteroid hormones ecdysone and 20-hydroxyecdysone play fundamental roles in insect postembryonic development and reproduction. Five cytochrome P450 monooxygenases (CYPs), encoded by Halloween genes, have been documented to be involved in the ecdysteroidogenesis in insect species of diverse orders such as Diptera, Lepidoptera and Orthoptera. Up to now, however, the involvement of the Halloween genes in ecdysteroid synthesis has not been confirmed in hemipteran insect species.

**Results:**

In the present paper, a Halloween gene *spook* (*Sfspo, Sfcyp307a1*) was cloned in the hemipteran *Sogatella furcifera*. SfSPO has three insect conserved P450 motifs, i.e., Helix-K, PERF and heme-binding motifs. Temporal and spatial expression patterns of *Sfspo* were evaluated by qPCR. *Sfspo* showed three expression peaks in late second-, third- and fourth-instar stages. In contrast, the expression levels were lower and formed three troughs in the newly-molted second-, third- and fourth-instar nymphs. On day 3 of the fourth-instar nymphs, *Sfspo* clearly had a high transcript level in the thorax where PGs were located. Dietary introduction of double-stranded RNA (dsRNA) of *Sfspo* into the second instars successfully knocked down the target gene, and greatly reduced expression level of *ecdysone receptor* (*EcR*) gene. Moreover, knockdown of *Sfspo* caused lethality and delayed development during nymphal stages. Furthermore, application of 20-hydroxyecdysone on *Sfspo-*dsRNA-exposed nymphs did not increase *Sfspo* expression, but could almost completely rescue *SfEcR* expression, and relieved the negative effects on nymphal survival and development.

**Conclusion:**

In *S. furcifera*, *Sfspo* was cloned and the conservation of SfSPO is valid. Thus, SfSPO is probably also involved in ecdysteroidogenesis for hemiptera.

## Background

20-Hydroxyecdyone (20E), an active form of ecdysteroid, regulates insect postembryonic development and reproduction. Because of the absence of the enzymes involving in squalene synthesis, insects cannot synthesize 20E *de novo,* and must obtain precursor sterols from their food [1], or their associated yeasts or fungi [2]. Rice planthoppers reportedly harbored yeast-like symbionts (YLSs), mainly in mycetocytes formed by abdominal fat body cells [3-8]. The YLSs synthesize ergosta-5,7,24(28)-trienol [9-12]. Ergosta-5,7,24(28)-trienol is then converted into cholesterol in planthoppers [10,11].

The ecdysteroid biosynthesis in the prothoracic glands (PGs) begins from conversion of cholesterol into 7-dehydrocholesterol (7dC), mediated by a Rieske oxygenase *Neverland*[13,14]. The conversion of 7dC into 2,22,25-trideoxyecdysone (ketodiol) is a series of hypothetical and unproven reactions, and is called ‘Black Box’ [15]. In *Drosophila melanogaster* and *Bombyx mori*, CYP307A1/A2 (SPOOK/SPOOKIER, SPO/SPOK) [16,17] and CYP6T3 [18] have been proven to be involved in the ‘Black Box’. Moreover, a paralog SPOOKIEST (SPOT, CYP307B1) was also found in CYP307 family [16,17]. RNAi mediated knockdown of *spok* in the PGs results in arrest of molting in *D. melanogaster*. Feeding two 3-oxo steroids, cholesta-4,7-diene-3,6-dione-14α-ol (Δ4-diketol) and 5β [H]cholesta-7-ene-3,6-dione-14a-ol (diketol), in the RNAi-treated larvae triggered molting, enhanced amounts of ecdysteroids and induced 20E inducible genes [19]. These results indicate that Δ4-diketol and diketol are components of the ecdysteroid biosynthetic pathway and lie downstream of a step catalyzed by SPOK/SPO. SPO- and/or SPOK-like proteins had found in other insect species in Diptera such as *Bemisia tabaci*[20], in Coleoptera such as the red flour beetle *Tribolium castaneum*[21], in Hymenoptera such as *Apis mellifera*[22], in Lepidoptera such as *Spodoptera littoralis*[23], *Manduca sexta*[17] and *Holcocerus hippophaecolus*[24], in Orthoptera such as *Schistocerca gregaria*[25], and in Hemiptera such as *Acyrthosiphon pisum*[26]. Up to now, however, involvement of SPO in ecdysteroidogenesis has not been confirmed in other insect species except *D. melanogaster* and *B. mori*.

Most actions of 20E are mediated through their nuclear receptor, the ecdysone receptor (EcR) and its heterodimer partner ultraspiracle. Mutations in and RNA interference (RNAi) against *EcR* cause phenotypic defects and lethality in *T. castaneum*[27], and in *Laodelphgax striatellus* and *Nilaparvata lugens*[28]. Moreover, *EcR* expression is regulated by ecdysteroids through a positive feedback loop directly [29] or indirectly in *D. melanogaster*[30].

The white-backed planthopper, *Sogatella furcifera*, was a secondary pest of rice before 1980s. However, since the mid-1980s, its population dramatically increased following a nationwide adoption of hybrid rice in China [31]. *S. furcifera* causes serious damage to rice plants by sucking the phloem sap and blocking the phloem vessels, and by acting as a virus vector to transmit Southern rice black-streaked dwarf virus [32-34]. Even though the complete genome sequence of *S. furcifera* is still unavailable, the transcriptome data have been published [35]. These data prompt us to identify and characterize the Halloween genes. Since dietary ingestion of double-stranded RNA (dsRNA) can effectively knock down target genes in planthoppers [36-39], our second goal in the present paper is to study the influence of the Halloween gene dsRNAs on the performance of *S. furcifera* nymphs, and the rescuing effects of 20E application on the negative influences of *spo*-dsRNA in the nymphs. Our results suggest that SfSPO play a critical role in ecdysteroidogenesis in *S. furcifera*.

## Results

### Molecular cloning and sequence analysis

**Figure 1:**
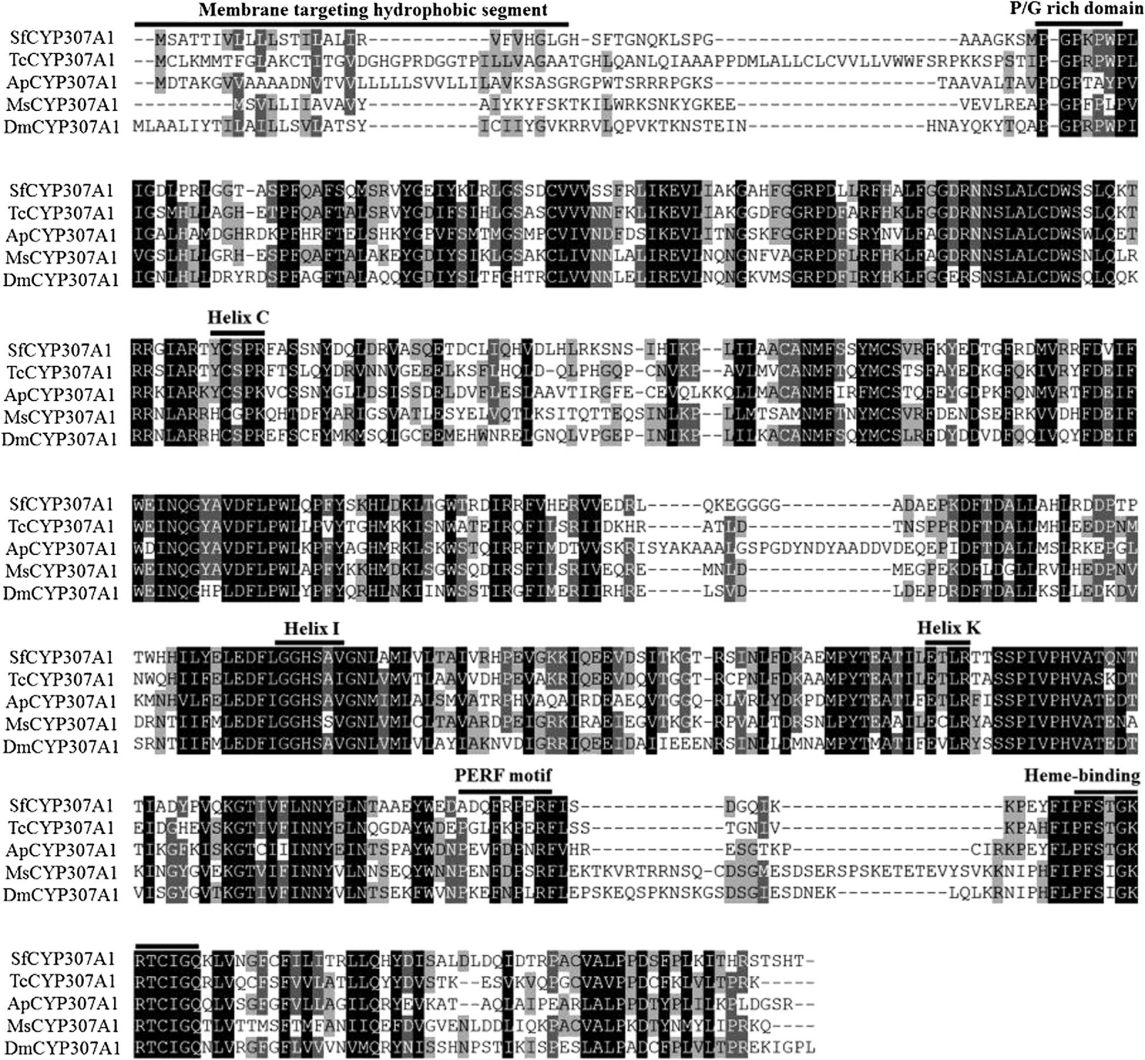
**Alignment of CYP307A1 (SPOOK, SPO) sequences from five insect species.** SPO originates from *Manduca sexta* (Ms) (ABI74778), *Drosophila melanogaster* (Dm) (NP_647975), *Tribolium castaneum* (Tc) (XP_969587), *Acyrthosiphon pisum* (Ap) (XP_001946295) and *Sogatella furcifera* (Sf), respectively. Amino acids with 100%, 80%, and 60% conservation are shaded in black, dark grey and light grey. The characteristic P450 structure, membrane targeting hydrophobic segment, P/G rich domain, Helix C, Helix I, Helix K, PERF motif and Heme-binding domain are shown in the figure.

Complete coding sequence of *S. furcifera* Halloween gene *Sfspo* (*spo, cyp307a1*) was obtained. Its open reading frame (ORF) encoded a putative protein with the length of 510 amino acid residue (Figure 1).

SPO sequence is similar to those from other insects. Insect CYPs have five insect conserved P450 motifs, i.e., WxxxR (Helix-C), GxE/DTT/S (Helix-I), ExxR (Helix-K), PxxFxPE/DRF (PERF motif) and PFxxGxRxCxG/A (heme-binding domain), where ‘x’ means any amino acid [40]. For SfSPO, Helix-C and Helix-I are not conserved. Helix-C had the amino acid sequence of H/YxxPR, and the amino acid sequence of Helix-I was GGHSA/V (Figure 1).

In insects, SPO belongs to CYP2 family. The N-terminus of SfSPO has one of the common characters in microsomal P450s, consisting many hydrophobic residues followed by a proline/glycine (P/G) rich region (Figure 1).

### Temporal and spatial transcript profiles

**Figure 2:**
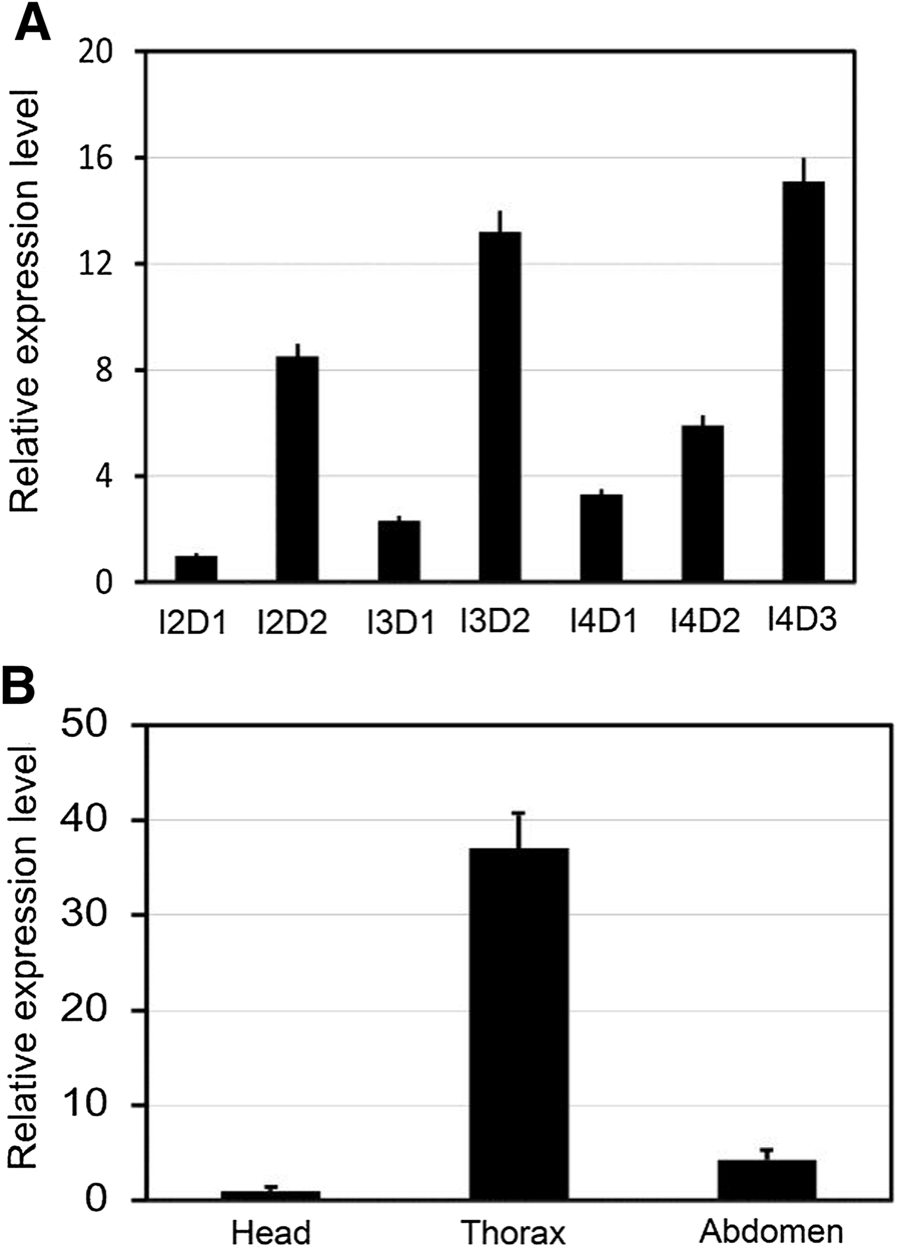
**Graphic representation of the relative *Sfspo *transcript levels measured in the whole bodies of second-, third- and fourth-instar (I2D1, I2D2, I3D1, I3D2, I4D1, I4D2 and I4D3) nymphs at 24 h intervals (A) and the head, thorax and abdomen of day 3 fourth-instar nymphs (B).** For each sample, 3 independent pools of 5–10 nymphs were measured in technical triplicate using qRT-PCR. The values were calculated using the 2^-ΔΔCt^ method. The relative expression levels were the ratios of relative copy numbers in individuals of specific developmental stage or specific body part to that in I2D1 or head. The columns represent averages with vertical bars indicating SE.

At our experiment temperature, *S. furcifera* second-, third- and fourth-instar nymphs lasted an average of 2.0, 2.0 and 3.0 days. *Sfspo* showed three expression peaks in day 2 of second-instar, day 2 of third-instar and day 3 of fourth-instar nymphs. In contrast, the expression levels were lower and formed three troughs in the newly-molted second-, third- and fourth-instar nymphs (Figure 2A).

The spatial distribution of *Sfspo* on day 3 of the fourth-instar nymphs was also tested using qPCR. *Sfspo* clearly had a high transcript level in the thorax where PGs were located. Moreover, trace amounts of transcripts were found in the head and abdomen (Figure 2B).

### Dietary ingestion of dsRNA on expression of *Sfspo* and *EcR* genes

**Figure 3:**
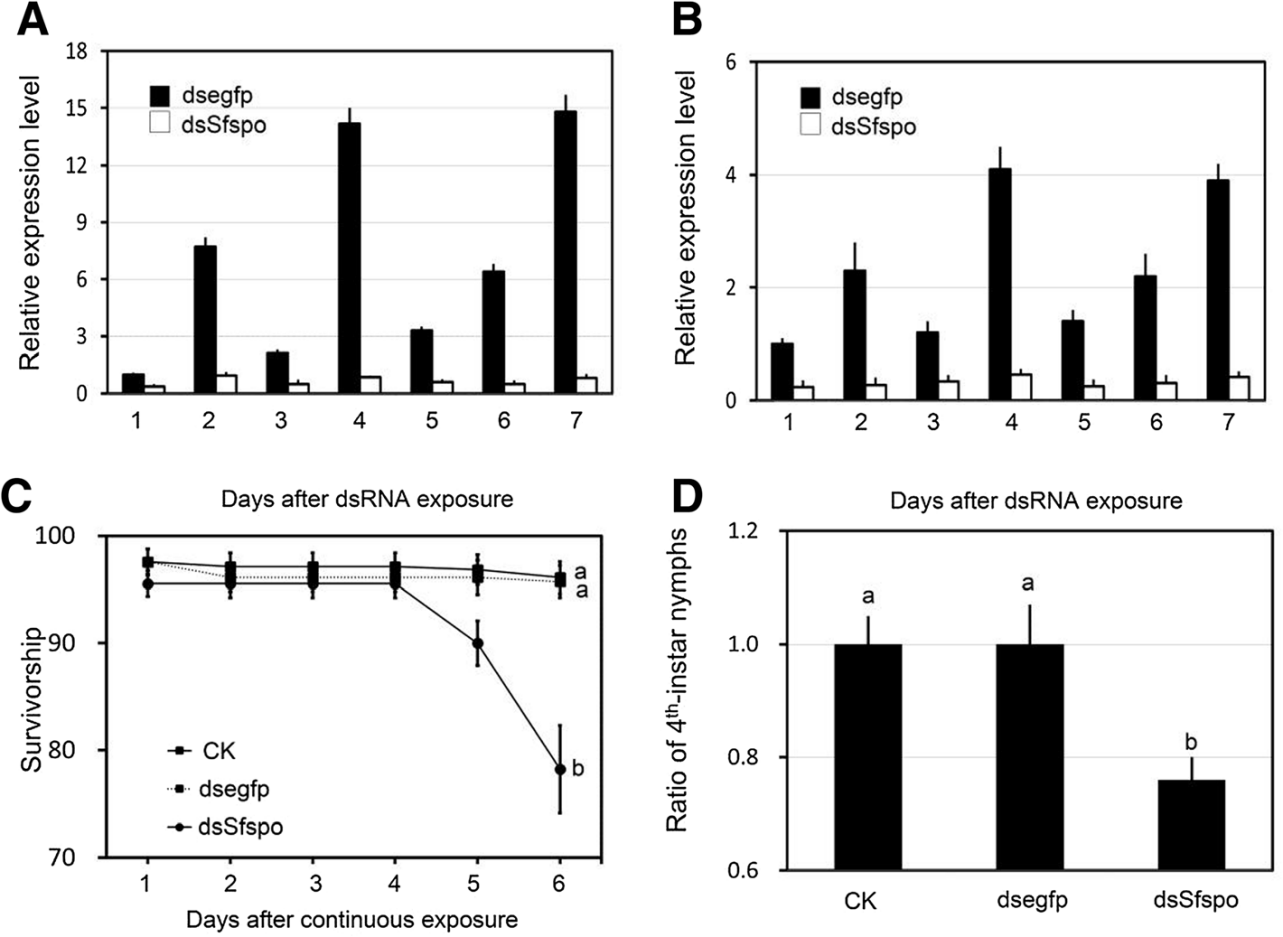
**Effects of dietary ingestion of ds*Sfspo *on the relative *Sfspo *(A) and *SfEcR *(B) transcript levels, survival (C) and development (D) of *L. striatellus *nymphs.** The nymphs were continuously ingested dsRNA from the second- through the fourth-instar stage. The relative transcript level for each sample was measured daily from 3 independent pools of 5–10 nymphs. The survival was calculated daily from 10 biological replicates, with each replicate of 10 individuals. The percentage of the fourth-instar was estimated from those in 10 biological replicates that survived through all experimental period. The values represent averages with vertical bars indicating SE, which topped with the same letters are not statistically significantly different at P = 0.05.

During 6 days of continuous exposure to dsRNA-contained diet and 1 day after experiment, mRNA abundance of *Sfspo* in the surviving nymphs was examined by q-PCR. The mRNA level of *Sfspo* in treated nymphs respectively reduced by 63.0%, 87.8%, 76.2%, 93.9%, 81.8%, 92.2% and 94.5%, respectively, comparing to that in ds*egfp*-exposed controls (Figure 3A). This indicated that the RNAi-mediated knockdown of *Sfspo* was successful.

Since SfSPO is expected to act in other genes in the same signaling pathway, the possible effect of *Sfspo* knockdown was examined on the transcript level of *SfEcR*, which was one of 20E heterodimeric nuclear receptors and was regulated by 20E through a positive feedback loop directly [29] or indirectly in *D. melanogaster*[30]. As expected, during 6 days of continuous exposure to dsRNA-contained diet and 1 day after experiment, *SfEcR* expression levels in nymphs decreased by 76.0%, 88.3%, 71.7%, 88.8%, 82.1%, 85.9% and 89.2% respectively, when compared with that in ds*egfp-*ingested planthoppers (Figure 3B).

### Effect of dsRNA on nymph survival

Six day ingestion of dsRNA-contained diet caused nymphal lethality. The mortality reached up to 20% in nymphs that had ingested ds*Sfspo*. In most cases, nymphs died during the period of ecdysis. In contrast, less than 5% of the planthoppers on normal or *egfp*-dsRNA-contained diets died (Figure 3C).

### Effects of dsRNA on nymph development

Six day period of continuous exposure to dsRNA-contained diet significantly delayed nymphal development. 100% of the nymphs on normal and *egfp*-dsRNA-contained diets became the fourth instars after experiment. In contrast, 24% of the individuals on *Sfspo-*dsRNA-contained diets remained in the third-instar (Figure 3D).

### Rescue experiment

**Figure 4:**
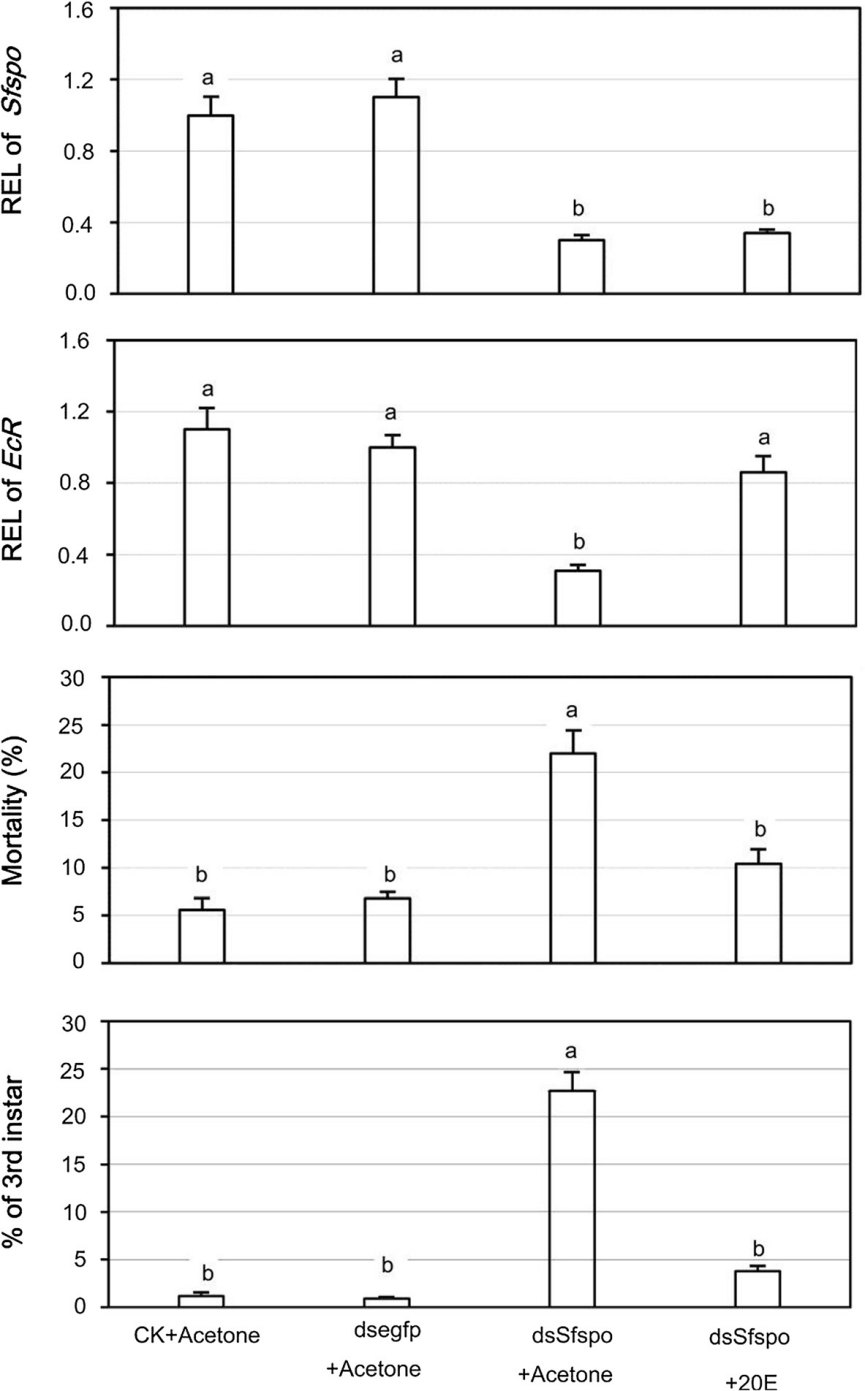
**Relative expression level (REL) of *spo *and *EcR *gene, mortality and percentage of third-instar nymphs in *S. furcifera *nymphs subjected to both ds*Sfspo *exposure and 20E application.** The nymphs were continuously ingested dsRNA from the second-instar through the early fourth-instar stage. Two-days after dsRNA exposure, the nymphs received 0.03 μL acetone or 300 pg of 20E in 0.03 μL acetone. The values represent averages with vertical bars indicating SE, which topped with the same letters are not statistically different at P = 0.05.

Application of 300 pg of 20E did not affect the expression level of *Sfspo*. In contrast, 20E application almost completely rescued *SfEcR* expression at mRNA level. Moreover, 20E application to *Sfspo*-dsRNA-exposed nymphs almost completely overcame the negative effects on the survival and the development (Figure 4).

## Discussion

Since the fundamental phenomena such as molting and metamorphosis are conserved during arthropod evolution, the Halloween genes are expected to be well conserved in insects [23,25,26,41-44], and in other arthropods [1,45]. In the present paper, the presence of *Sfspo* was demonstrated in *S. furcifera*. The primary structure of SfSPO has three insect conserved P450 motifs, i.e., Helix-K, PERF and heme-binding motifs. Similar structural characters have been documented in SPO- and SPOK-like proteins from other insect species of diverse orders such as Diptera [16,17,20], Coleoptera [21], Hymenoptera [22], Lepidoptera [17,23], Orthoptera [25], and Hemiptera [26]. The N-terminus of SfSPO has one of the common characters in microsomal P450s, consisting many hydrophobic residues followed by a proline/glycine (P/G) rich region. Consistent with the structural features, SPO is detected in endoplasmic reticulum (ER) when the corresponding gene is transfected to *Drosophila* S2 cells [17,46]. Moreover, *Sfspo* showed three expression peaks in late second-, third- and fourth-instar stages. In contrast, the expression levels were lower and formed three troughs in the newly-molted second-, third- and fourth-instar nymphs. In the fourth-instar nymphs of the brown planthopper *N. lugens*[47] and in the sixth-instar larvae of a lepidopteran species *S. littoralis*, the level of ecdysteroid showed a peak in the later instar stage. In *D. melanogaster* larval stage, expression patterns of *Dmspo* gene undergoes dramatic fluctuations, consistent with circulating ecdysteroid quantity in the haemolymph: being high in late seconds, low in early third and high in late thirds [17]. Furthermore, we found in this study that *Sfspo* clearly had a high transcript level in the thorax where PGs were located. Similarly, *Dmspo* is expressed primarily in the PG cells of the ring gland in larval and adult stages [17]. Thus, the structural features and temporal and spatial expression patterns suggest that SfSPO might be involved in the ecdysteroidogenesis in *S*. *furcifera*.

The suggestion is further confirmed by three lines of experimental evidence in the present paper. Firstly, RNAi-mediated knockdown of *Sfspo* in *S*. *furcifera* reduced the expression level of *SfEcR* at the mRNA level. In other insect species, mutations in or RNAi against the Halloween enzymes caused a decrease in ecdysteroid titers [23,25,26,44,46,48-51]. Moreover, the expression of *EcR* gene was regulated by ecdysteroids through a positive feedback loop in *D. melanogaster*[29,52]. Accordingly, it can be hypothesized that RNAi-mediated knockdown of *Sfspo* negatively affects ecdysteroidogenesis in *S*. *furcifera*, and subsequently down-regulated *SfEcR* expression in *S*. *furcifera*. Consistent with the hypothesis, our rescue experiment revealed that 20E application almost completely rescued *SfEcR* expression in nymphs that had ingested ds*Sfcyp307a1.*

The second line of experimental evidence is that RNAi-mediated knockdown of *Sfspo* in *S*. *furcifera* caused phenotypic defects similar to insects whose ecdysteroid synthesis was disturbed or whose ecdysteroid-mediated signaling had been inhibited [53,54]. In the present paper, we found that ingestion of ds*Sfspo* caused nymphal lethality and developmental delay. Since the average second- and third-instar periods of the nymphs in our experimental conditions was respectively about 2 days and the deaths mainly occurred in the sixth day after dsRNA exposure, it means that the nymphs died during the third ecdysis. In fact, we also observed many abnormal and lethal ecdysis individuals on *Sfspo*-dsRNA contained diet, whereas most of the larvae on control normally molted. Similar phenomena have been observed in other two rice planthoppers, *L. striatellus* and *N. lugens*, in which silencing of *EcR* expression by *in vivo* RNAi to inhibit ecdysteroid-mediated signaling generated phenotypic defects in molting and resulted in lethality in most of the treated nymphs. Intriguingly, apparent wing defects in morphogenesis and melanization occurred in *L. striatellus* nymphs subjected to ds*EcR* microinjection [54].

It has long been known that topical application of 20E could trigger physiological response such as regulation of diapause in the fourth-instar planthopper nymphs [55]. In the present paper, we tested whether 20E could rescue the negative effects of *Sfspo*-dsRNA ingestion on nymphs. Our results revealed that 20E application to *Sfspo*-dsRNA-exposed nymphs almost completely relieved the negative effects on the survival and the development. Thus, we provided the third line of evidence to support the suggestion that SfSPO plays critical roles in ecdysteroidogenesis in *S*. *furcifera.*

## Conclusions

In the present paper, we cloned *Sfspo* and found that the conservation of SfSPO is valid in *S. furcifera*. Thus, SfSPO is probably also involved in ecdysteroidogenesis for hemiptera.

## Methods

### Insect culture and chemicals

*S. furcifera* adults were collected from Nanjing (32.0° N, 118.5° E), Jiangsu Province in China in 2010. The strain has been reared routinely on rice (*Oryza sativa*), in an insectary under controlled temperature (28 ± 1°C), photoperiod (16 h light/8 h dark) and relative humidity (more than 80%) since then, with wild stock injections every summer. Rice variety (Taichung Native 1) was grown in soil at 30–35°C under a long day photoperiod (16 h light/8 h dark) in a growth incubator. The planthoppers were transferred to fresh seedlings every 10–14 days to assure sufficient nutrition.

At laboratory reared by above protocol, *S. furcifera* eggs hatched into nymphs within 7 days. Nymphs went through 5 instars, with the average periods of the first-, second-, third-, fourth- and fifth-instar stages of 2.5, 2.0, 2.0, 3.0 and 3.0 days, respectively. Upon reaching full size, the fifth-instar nymphs emerged as adults.

20E was purchased from Sigma, and was purified by reverse-phase HPLC before experiments.

### Sequence assembly and homology searches

Raw nucleotide reads of *S. furcifera* were downloaded from the NCBI Sequence Read Archive (SRA) database with its accession number SRP009194, and assembled into unigenes using Trinity software [56]. The annotated SPO from 4 representative insect species *A. pisum*, *T. castaneum*, *M. sexta* and *D. melanogaster* were downloaded from NCBI reference sequences (RefSeq) database. These protein sequences were used for TBLASTN searches of *S. furcifera* transcriptome data to identify hits at a cutoff E-value of 1.0^-5^. The nucleotide sequences of hits resulting from initial searches were annotated by blasting (BLASTX, e-values < 10^-5^) against a local protein database containing NCBI non-redundant proteins.

### Molecular cloning

Total RNA was extracted from the fourth-instar nymphs using TRIzol reagent according to the manufacturer’s instructions (Invitrogen), and was treated for 30 min at 37°C with RNase free DNase I (Ambion, Austin, TX) to eliminate traces of chromosomal DNA. The purity and amount of RNA were determined by NanoDrop ND-1000 spectrophotometer (Nanodrop Technologies, Rockland, DE, USA). First-strand cDNA was synthesized from the total RNA using the reverse transcriptase (M-MLV RT) (Takara Bio., Dalian, China) and an oligo (dT)_18_ primer, and was used as a template for polymerase chain reaction (PCR) to authenticate the sequences of the selected unigenes. The primers based on the sequences were designed using Primer3 software [57]. Once initial *Sfspo* unigenes were authenticated, they were aligned to the full cDNA sequence of the gene from the 4 representative insect species mentioned above. Some short sequence gaps between two aligned unigenes were found. Specific primers were designed based on the two unigenes between each gap, and the gaps were filled by PCR. The final cDNA sequence was authenticated using the primers listed in Table 1. Thermal cycling conditions were 94°C for 5 min, followed by 35 cycles of 94°C for 30 sec, 55°C for 45 sec and 72°C for 3 min. The last cycle was followed by final extension at 72°C for 10 min. Each 50 μL PCR reaction contained 2 μL of cDNA template, 5 μL of 10× LA Taq buffer (Mg^2+^ Free), 4 μL of MgCl_2_ (25 mM), 4 μL of dNTP mixture (2.5 mM/each), 1 μL of forward and 1 μL of reverse primers (10 μM), 0.5 μL of LA Taq polymerase (Takara Bio.) (5 U/μL) and 32.5 μL of double distilled H_2_O.

**Table 1:** Primers used in RT-PCR, 5’ and 3’ RACE, synthesizing dsRNA, and performing qRT-PCR

**Primer**	**Sequence (5’ to 3’)**	**Amplicon size (bp)**
Primers used in RT-PCR		
spoFp	ACGGCCAGTCCATTTCAG	344
spoRp	TGTTGGATGAGGCAGTCG	
Primers used in 5’-RACE		
spoGSP	GATGCCAAGTGAGGGTGGGATC	
spoNGSP	TCGGTGAAGTCTTTGGGCTCGG	
Primers used in 3’RACE		
spoGSP	ACGCGACATTCGCCGCTTTGTG	
spoNGSP	CGATGCGCTGCTCGCTCACCTT	
Primers used in PCR for End to End		
spoFp	CGTCGTGAACACCCTTAT	2090
spoRp	GCCCGGTACACTATTATCTT	
Synthesizing the dsRNAs		
spoFd	CCCTCACTTGGCATCAC	415
spoRd	TCGGGTTTCTTTATTTGTC	
egfpup	AAGTTCAGCGTGTCCG	414
egfpdown	CTTGCCGTAGTTCCAC	
Performing the qPCR		
spoFq	CAACTCCATACACATCAAGCCACTG	119
spoRq	ACCGACGCACCATATCTCTGAAC	
EcRFq	AATGAGTTCGAGCACCCTAGCGAA	129
EcRRq	AATGGTGATTTCGGTGATGTGGCG	
RPL9Fq	TGTGTGACCACCGAGAACAACTCA	131
RPL9Rq	ACGATGAGCTCGTCCTTCTGCTTT	
ARFFq	CACAATATCACCGACTTTGGGATTC	141
ARFRq	CAGATCAGACCGTCCGTACTCTC	

The 5’- and 3’-RACE Ready cDNA were synthesized following the manufacturer’s instructions, primed by oligo (dT) primer and the SMART II A oligonucleotide using the SMARTer RACE cDNA amplification kit (Takara Bio.). Antisense and sense gene-specific primers (Table 1) corresponding to the 5’- and 3’-end of the sequence obtained above, and the universal primers in the SMARTer RACE kit (Takara Bio.) were used to amplify the 5’-end and the 3’-end. The components of reaction have been described above. Thermal cycling conditions were 94°C for 3 min; followed by 5 cycles of 94°C for 30 sec, 72°C for 5 min; and another 5 cycles of 94°C for 30 sec, 70°C for 30 sec, 72°C for 5 min; and followed by 25 cycles of 94°C for 30 sec, 68°C for 30 sec, 72°C for 5 min. The last cycle was followed by final extension at 72°C for 10 min.

The amplified product was separated by 1.2% agarose gel and purified with Wizard DNA Gel Extraction Kit (Promega, Madison, Wis., USA), and then cloned into pGEM-T easy vector (Promega). Several independent subclones were sequenced on an Applied Biosystems 3730 automated sequencer (Applied Biosystems, Foster City, Calif., USA) from both directions.

After full-length cDNA was obtained, we designed primers (Table 1) to verify the complete ORF with the same PCR conditions outlined above. ORF was predicted using the editseq program of DNAStar (http://www.dnastar.com) and the features of the protein were determined by TargetP. The resulting sequence was submitted to GenBank (KC579454). The annotated SPO-like proteins from the 4 representative insect species mentioned above were aligned with the predicted LsSPOK using ClustalW2.1 [58].

### Preparation of dsRNA

A 415 bp cDNA sequence of *Sfspo* and a 414 bp fragment of enhanced green fluorescent protein gene *egfp* (control) were individually subcloned into pEASY-T3 vector (TransGen Biotech, Beijing, China), and the diluted plasmids were used as templates for amplification of these target sequences by PCR, using specific primers (Table 1) conjugated with the T7 RNA polymerase promoter (5’-taatacgactcactataggg-3’) and the PCR conditions described above. The PCR products were purified with Wizard H SV Gel (Promega) and used as templates for dsRNA synthesis with the T7 Ribomax TM Express RNAi System, according to the manufacturer’s instructions (Promega). The reaction products were treated with RNase and DNase I to degrade single-strand RNA and DNA template, respectively, at 37°C for one hour, following manufacturer’s directions. The synthesized dsRNA was isopropanol precipitated, resuspended in Nuclease-free water, and quantified by a spectrophotometer (NanoDrop TM 1000) at 260 nm. The purity and integrity were determined by agarose gel electrophoresis. The dsRNA stocks can be stored for several weeks at -80°C until use.

### Bioassay

Previously reported dietary dsRNA-introducing procedure [38,39] was used, with small modifications. Briefly, glass cylinders, 12 cm in length and 2.8 cm in internal diameter, were used as feeding chambers. Twenty first-instar nymphs were carefully transferred into each chamber and pre-reared for one day to the second-instar stage, on liquid artificial diet (according to Dr. Fu et al. [59]) between two layers of stretched Parafim M (Pechiney Plastic Packaging Company, Chicago, IL, USA) that was placed at both ends of the chamber. The artificial diet containing one of the dsRNAs at the concentration of 0.5 mg/ml [38,39] were then used to feed the second-instar nymphs. The diet was changed and dead nymphs were removed daily.

Two experiments were carried out. The first had three treatments including non-dsRNA diet (blank control), ds*egfp* diet (negative control) and ds*Sfspo* diet. The experiment lasted for 6 days. The second bioassay was a rescue experiment. Since topical application of 300 pg of 20E was enough to trigger physiological response in the fourth-instar planthopper nymphs [55], 300 pg of 20E was used in the second bioassay. After exposed to dsRNA for 2 days, the nymphs were anesthetized with carbon dioxide. A 0.03 μL aliquot of acetone with or without 300 pg of 20E was topically applied to the dorsal thoracic surface of the nymphs with a 10-μL microsyringe connected to a microapplicator (Hamilton Company, Reno, NV). And then, the nymphs were continuously exposed to dsRNA for another 4 days. There were four treatments including: (1) nymphs on non-dsRNA diet and applied acetone (blank control); (2) nymphs on ds*egfp* diet and applied acetone (negative control); (3) nymphs on ds*Sfspo* diet and applied acetone; (4) nymphs on ds*Sfspo* diet and applied 20E. All treatments in both experiments were replicated 25 times (25 chambers), and a total of 250 nymphs in each treatment were used (100 nymphs for bioassays and 150 nymphs for q-PCR).

Mortality was recorded daily. The surviving nymphs after bioassay were collected and frozen. The instars of the surviving nymphs were identified by head capsule width and the number of rhinaira (sensilla clusters) bearing on the pedicle of the antennae [60,61].

### Real-time quantitative PCR

Total RNA samples were prepared from the whole bodies of the of second-, third- and fourth-instar (I2D1, I2D2, I3D1, I3D2, I4D1, I4D2 and I4D3) nymphs, from the head, thorax and abdomen of I4D3 nymphs on rice, and from nymphs subjected to 6-day’s bioassays, using SV Total RNA Isolation System Kit (Promega). Each sample contained 10 nymphs and repeated in biological triplicate. Purified RNA was subjected to DNase I to remove any residual DNA according to the manufacturer’s instructions. In a preliminary experiment, we estimated the expression stability of four house-keeping genes (*Actin*; *ADP-ribosylation factor*, *ARF*; *ribosomal protein RPL9*; *translation elongation factor 1α EF1α*), and found that *ARF* and *RPL9* were the most stable house-keeping genes and selected as internal controls. The primers of the Halloween genes *Sfspo*, *EcR* gene, *ARF* and *RPL9* were designed with Beacon Designer 7 (Table 1). Putative mRNA abundance of *Sfspo* the Halloween and *EcR* genes in each nymphal sample was estimated by qPCR using SYBR Premix Ex Taq™ (Perfect Real Time) (Takara Bio.) and ABI 7500 Real-Time PCR System (Applied Biosystems) according to the manufacturer’s instruction. The reaction mixture consisted of 2 μL of cDNA template (corresponding to 50 ng of the starting amount of RNA), 10 μL of SYBR Premix Ex Taq (Takara Bio.), 1 μL of forward primer (10 μM), 1 μL of reverse primer (10 μM), 0.4 μL of Rox Reference Dye (50×) in a final reaction volume of 20 μL. A reverse transcription negative control (without reverse transcriptase) and a non-template negative control were included for each primer set to confirm the absence of genomic DNA and to check for primer-dimer or contamination in the reactions, respectively. The following standard qPCR protocol was used: denaturing at 95°C for 30 sec, followed by 40 cycles of 95°C for 5 sec and 60°C for 34 sec. After amplification, the melting curves were determined by heating the sample up to 95°C for 15 sec, followed by cooling down to 60°C for 1 min, and heating the samples to 95°C for 15 sec.

The generation of specific PCR products was confirmed by sequencing and gel electrophoresis. Each primer pair was tested with a 10-fold logarithmic dilution of a cDNA mixture to generate a linear standard curve (crossing point CP plotted vs. log of template concentration), which was used to calculate the primer pair efficiency. All experiments were repeated in technical triplicate. Data were analyzed by the 2^-ΔΔCt^ method [62], using the geometric mean of *ARF1* and *RP18* for normalization according to the strategy described previously [62,63].

### Data analysis

The data were given as means ± SE, and were analyzed by ANOVAs or a repeated measures ANOVA followed by the Tukey-Kramer test, using SPSS for Windows (SPSS, Chicago, IL, USA).

## Abbreviations

PCR: Polymerase chain reaction; RT-PCR: Reverse transcriptase PCR; qRT-PCR: Quantitative real-time PCR; cDNA: Complementary DNA; CYP: Cytochrome P450 monooxygenase; dsRNA: Double-stranded RNA; EcR: Ecdysone receptor; E: Ecdysone; 20E: 20-Hydroxyecdysone; YLS: Yeast-like symbionts; RNAi: RNA interference; ORF: Open reading frame; ML: Maximum-likelihood; SE: Standard error; ANOVA: Analysis of variance.

## Competing interests

The authors declare that they have no competing interests.

## Authors’ contributions

SJ and PJW performed most of the experimental procedures, and data analysis. LTZ and LLM performed partial experiments, assisted in manuscript revising and provided helpful discussions. GQL wrote the manuscript, conceived and supervised the research. All authors read and approved the final manuscript.
